# Angle and Context Free Grammar Based Precarious Node Detection and Secure Data Transmission in MANETs

**DOI:** 10.1155/2016/3596345

**Published:** 2016-01-04

**Authors:** Anitha Veerasamy, Srinivasa Rao Madane, K. Sivakumar, Audithan Sivaraman

**Affiliations:** ^1^Department of Information Technology, Anna University, Chennai, Tamil Nadu 600025, India; ^2^Department of Computer Science and Engineering, Adhiparasakthi College of Engineering, Vellore, Tamil Nadu 603319, India; ^3^Department of Computer Science, College of Computer Science, King Khalid University, Abha 62529, Saudi Arabia; ^4^Department of Electronics and Communication Engineering, PRIST University, Thanjavur, Tamil Nadu 613403, India

## Abstract

Growing attractiveness of Mobile Ad Hoc Networks (MANETs), its features, and usage has led to the launching of threats and attacks to bring negative consequences in the society. The typical features of MANETs, especially with dynamic topology and open wireless medium, may leave MANETs vulnerable. Trust management using uncertain reasoning scheme has previously attempted to solve this problem. However, it produces additional overhead while securing the network. Hence, a Location and Trust-based secure communication scheme (L&TS) is proposed to overcome this limitation. Since the design securing requires more than two data algorithms, the cost of the system goes up. Another mechanism proposed in this paper, Angle and Context Free Grammar (ACFG) based precarious node elimination and secure communication in MANETs, intends to secure data transmission and detect precarious nodes in a MANET at a comparatively lower cost. The Elliptic Curve function is used to isolate a malicious node, thereby incorporating secure data transfer. Simulation results show that the dynamic estimation of the metrics improves throughput by 26% in L&TS when compared to the TMUR. ACFG achieves 33% and 51% throughput increase when compared to L&TS and TMUR mechanisms, respectively.

## 1. Introduction

**Figure 1:**
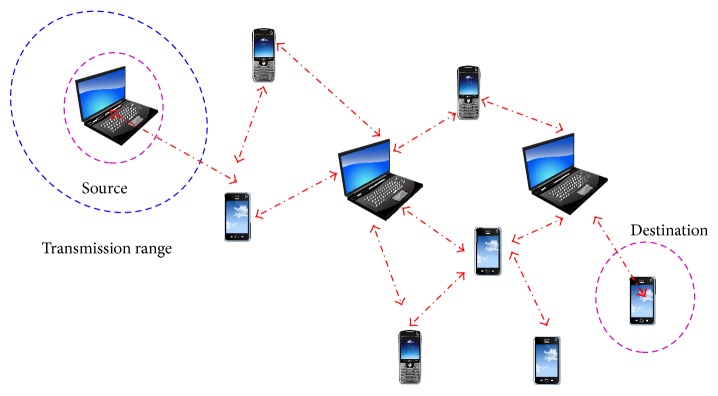
Mobile Ad Hoc Networks.

Mobile Ad Hoc Networks (MANETs) embrace various computational nodes that can communicate with one another within a specified wireless range. The most favoured feature of MANETs is their capability to allow communication during node mobility. However, the shared wireless medium of MANET facilitates inactive and adversarial eavesdropping on data communications, where adversaries can start various overwhelming attack on the network. Many protocols have been designed for protecting the wireless communication but do not grant significance in privacy protection and leave mobile nodes to be noticeable by wireless analysis. Secure data transmission in MANET is thus a very challenging task. An example MANET is shown in Figure 1.

To overcome this problem, two strategies are proposed and evaluated in this paper, Location and Trust-based secure communication scheme (L&TS) and Angle and Context Free Grammar (ACFG) based precarious node detection and secure communication in MANETS.

Firstly, a Location and Trust-based secure communication scheme assigns algorithms for data integrity based on how far the nodes are located from one another. The next hop is selected based on the trust. A trust value is calculated based on the previous network operations for effective next hop selection, which makes this scheme work efficiently even under high mobility conditions. A design limitation identified in this scheme has motivated us in the design of ACFG scheme for MANETs.

In ACFG scheme, the next hop is selected based on a node's angle and a node's left most and right most derivations. There are three levels of assessment: first the node's location is assessed using the angle method, followed by the CFG to detect which node among the neighbors has faked the location; the last confirmation using an elliptic cryptography function is achieved to publish that a node is malicious. In this scheme, there is no requirement for extraordinary nodes for the localization process or for other special purposes. Hence, it provides better performance when compared to L&TS mechanism. The organization of the paper is, thus, related works following Introduction, the proposed methods, and simulation analysis.

## 2. Related Work 

The works related to the mechanism proposed here are broadly classified based on security, location, and Angle and Context Free Grammar (CFG) used as estimation parameters while routing.

### 2.1. Security

Many protocols have improved security using different aspects in the literature. The ad hoc networks are classified into three types: open, managed-open, and managed-hostile, dissimilar in the safety requirement. SPAAR is one protocol that aims to provide security in a* managed-hostile *environment, which is described as a MANET created using military nodes in a battle situation [1]. Secure Routing Protocol (SRP) [2], on the other hand, needs a security association across end nodes assuring that they can differentiate and drop reply messages giving any fake information or even stop receiving the same. This is achieved by employing and using a shared secret to the main routing protocol, for example, AODV. The trust based routing protocols [3, 4] are assigned trusted values and the data are routed only through trusted nodes.

Selection of security scheme for every data packet and management of the same is a tedious and expensive process [5]. In the Authenticated Anonymous Secure Routing (AASR) [6] the RREQ packets are legitimated using group signatures and to preserve vigorous attacks from unveiling the node distinctiveness. The key-encrypted onion routing containing a route secret verification message prevents the nodes from avoiding misinterpretation of a genuine intention. Provision of high anonymity and security is considered an advantage. However, there is enormous packet delay during data transmission. The sensors' decision reports are used to discover the malicious nodes and estimate their attack behaviour. The detection procedure is analyzed using the entropy-defined trust model [7].

### 2.2. Location

Localization verification technique [8] depends on the received signal strength. A node confirms the truthfulness of the other node by predicting its next geographical localizations and checks the similarity to the actual localizations found. A node confirms the truthfulness of the other node by predicting its next geographical localizations and checks the similarity to the actual localizations found in Efficient Mobility Based Localization (EMBL) [9]. During the localization method, each node predicts its future mobility pattern according to its past known location information. However, there is poor accuracy in predicting the real and estimated node positions, and distances from a reference node. Ordinary nodes among anchor nodes and unknown nodes build a shortest path by greedy approach [10]. This shortest path is approximately a straight line between unknown nodes and reference nodes. The disadvantage of this algorithm is the poor accuracy when there are too many routers.

In Modified Parametric Location Identification (MPLI) [11], location is identified separately using the *x* and *y* coordinates, angle of arrival, time, distance, and circular region quadrants. It also provides timely updates of positions to make the routing more robust and position aware to avoid data losses and connection termination due to mobility. Multihop Localization Algorithm (MLA) [12] has five steps as follows: (1) achieving neighbor distances, (2) calculating reference node distances (unidentified nodes can estimate the total distances to nodes with the data obtained from step 1, and they decide the shortest paths to anchor), (3) choosing the reference nodes, (4) acquiring angles, and (5) fitting the shortest path to straight line.

### 2.3. Angle

Decoupled Maximum Likelihood (DEML) angle estimator determines the angle of arrival [13]. The DEML estimator is no longer asymptotically statistically proficient. Angular Routing Protocol (ARP) [14] based on position that uses an improved geographic forwarding to route packets to the destination. The geographic forwarding fails, at a time used by the angle-based forwarding method. It does not require establishing routes. The indefinite node estimates its angle to each of the three reference nodes, based on these angles, and the positions of the reference nodes (that form a triangle), and computes its own position using simple trigonometrical relationships [15]. The triangular zone [16] is used to reduce the route searching space. This mechanism avoids huge routing traffic and collision.

The angle is found based on slope of line. Slope values are found in all neighboring nodes [17]. The angles between unidentified node and several fixed nodes are used in the AOA (angle of arrival) [18] to evaluate the position, which is a little costly to perform. Orthomorphic Analyst *k*-Nearest Neighbor method [19] detects the intrusion activity based on the traffic intensity at inner boundary instance within the communication MANETs. Angle based distance is measured between the node points for easy detection of traffic creating nodes. It measures how far each pair of mobile nodes is and evaluates correct angle of position within the inner boundary.

### 2.4. CFG

One-time authentication information [20] discussed structured and unstructured techniques for generating strings and analyses of the difficulty of guessing strings in such a language. This authentication information is used for the generation of one-time passwords. This one-time authentication information is still inclined to operate in the middle attacks. Probabilistic Context Free Grammars (PCFGs) [21] recognized events from raw sensor network measurements. It derived a brief probabilistic Context Free Grammar from the known examining data. A metric depending on Bayesian formula for maximizing grammar, a posteriori probability given the training data, is used here. Advantages of the properties are the chunk and merge operations.

Translate natural language sentence into database query NLDBI (Natural Language Interface to a Database) structure [22] including its probabilistic Context Free Grammar, it is used to construct the parse tree, and this algorithm calculates the probabilities. CFG has a formalization capability in describing most sentence structures and so well formed that efficient sentence parser.

Trust management system enhances the protection in MANETs [18]; the trust model has trust from direct and indirect observations. In direct observation, the trust value is derived using Bayesian inference; it is a type of uncertain reasoning where the full probability model can be defined. In indirect observation, the trust value is derived using the Dempster-Shafer theory; it is another type of uncertain reasoning where the proposition of interest can be derived.

This method separates data packets and control packets and mitigates factors that cause packet losses. After examination of the trust value, there is a possibility for the inside attackers to conquer the other nodes. In such a situation, the Location and Trust-based secure communication scheme is used to provide security in MANETs.

## 3. Location and Trust-Based Secure Communication Scheme (L&TS) 

The proposed scheme provides a technique to manage and use different security schemes in a single MANET simultaneously. It uses the security algorithms available as security providers based on how far the nodes are located from the corresponding destination nodes. The security schemes are known in prior to the nodes operating in the MANET. Each node chooses its own security algorithm based on how far the destination is located and sends the data to the destination. This is performed with an understanding that the farthest nodes or the nodes that require multihop transmission require higher security than nodes that are closer to each other and are capable of direct transmission.

### 3.1. Working of the Scheme

**Figure 2:**
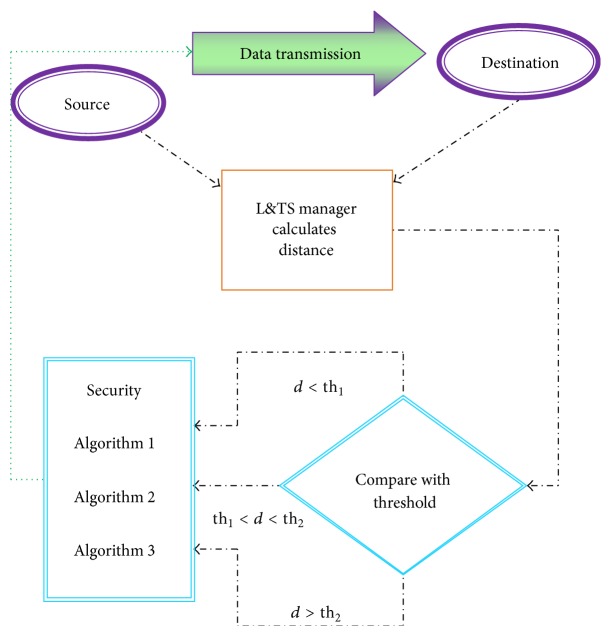
Working of the L&TS scheme.

The actual working of the scheme can be divided into the following processes: Data Collection, L&TS Management, Selection of Security Algorithm, and Data Transmission. The architectural working of the L&TS in MANETs is described in Figure 2.

#### 3.1.1. Data Collection

Each node in the MANET sends a route request RREQ message along with the location information in order to transmit data to its destination. In return, it receives a route reply RREP message that also contains the location information of the destination. The information about the most recent transmissions for that node is collected from every node as well.

#### 3.1.2. L&TS Management

An arbitrary manager called the L&TS manager processes the information collected. Based on the location information of the source and destination the distance between them is calculated dynamically and compared with threshold values th_1_, th_2_, and th_3_ (in this case) and it can go up to th_*i*_. The trust values *T*(*n*) are also assigned based on the most recent transmissions.

#### 3.1.3. Selection of Security Algorithm

A number of security algorithms (i.e., RSA + MD5; HMAC + MD5; and HMAC + SHA-1) are stored in a database and based on the comparison of the L&TS manager, a particular transmission is assigned Algorithm *i*. For example, if *d* value is less than th_1_ then Algorithm 1 is selected; if it is less than th_2_, then Algorithm 2 is selected, and so on up to Algorithm *i*. It is significant to mention that Algorithm 1 has lower security standard than Algorithm 2.

#### 3.1.4. Data Transmission

The data transmission is carried out based on the trust values *T*(*n*) of the nodes in the network through shortest path distance. If the source and destination are within the range of each other, then direct communication is performed. The threshold values are stable yet the mobility of the nodes alters the type of security algorithm used when the *d* value switches from one threshold value to another. This makes the transmission security switch from Algorithm 1 to Algorithm 2. This is a well-informed process so the destination alone knows which algorithm is used and when.

**Figure 3:**
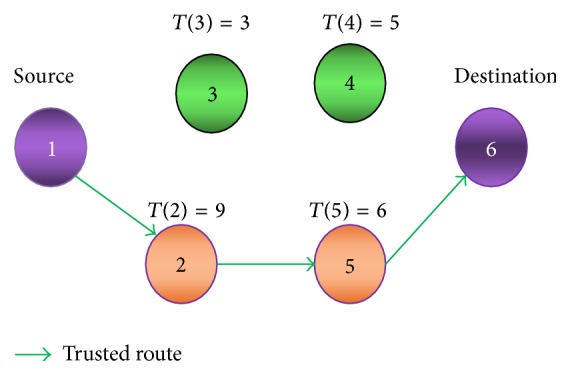
Trust route formation.

Figure 3 shows the Trusted Route obtained from the history of usage of a node for previous network operations. A node that is newly introduced into the MANETs is assigned an optimum history value *H* by authenticated persons. For a normal node, as the network operations are performed in the system, the value of *T*(*n*) is incremented relatively by the nodes that communicate to it. If the quality of service (QoS) of the node is diminishing then the value of *T*(*n*) is decremented. Based on the value of *T*(*n*) at the instant *i*, the routing to the destination along with the selected security scheme is performed. Distrusted nodes are not preferred for the routing operations; this provides security during routing.

#### 3.1.5. Result Notification

In this phase, the security is provided based on the destination location from the source. The trust value *T* is estimated from history of usage of previous network communication process. It works well under high mobility.

### 3.2. Limitation of L&TS

The forwarding node is selected based on the history of previous transmission. There exists a drawback in L&TS that has been identified in this section. The cost of this system is considerably high.

## 4. Angle and Context Free Grammar (ACFG) Based Precarious Node Detection and Secure Data Transmission

Trust management using uncertain reasoning method has higher delay and packet loss. Even after the evaluation of the trust value, there is a possibility for the insider attackers to ruin the nodes. Therefore, there is an absolute need to introduce a novel method to improve the security. Pertaining to this concern, an Angle and CFG based precarious node detection and secure data transmission mechanism is proposed in MANETs. In this scheme, the precarious node detection uses Angle and CFG method (Levels 1 and 2) and secure data transmission after publishing malicious nodes using Elliptic Curve Cryptography (Level-3).

### 4.1. Forwarding Node Selection

If a source node (*S*) likes to send a packet to the destination (*D*), the source selects a next hop (*N*) from its neighbor table. The next hop selects the closest node to the destination among the neighboring nodes of the source node. The next hop node is selected based on the minimum spanning tree shortest path algorithm.

### 4.2. Neighbor Angle Computation

The source selects the neighbor node *N* and collects their surrounding neighbor nodes and then computes the angles of the neighbor nodes.

**Figure 4:**
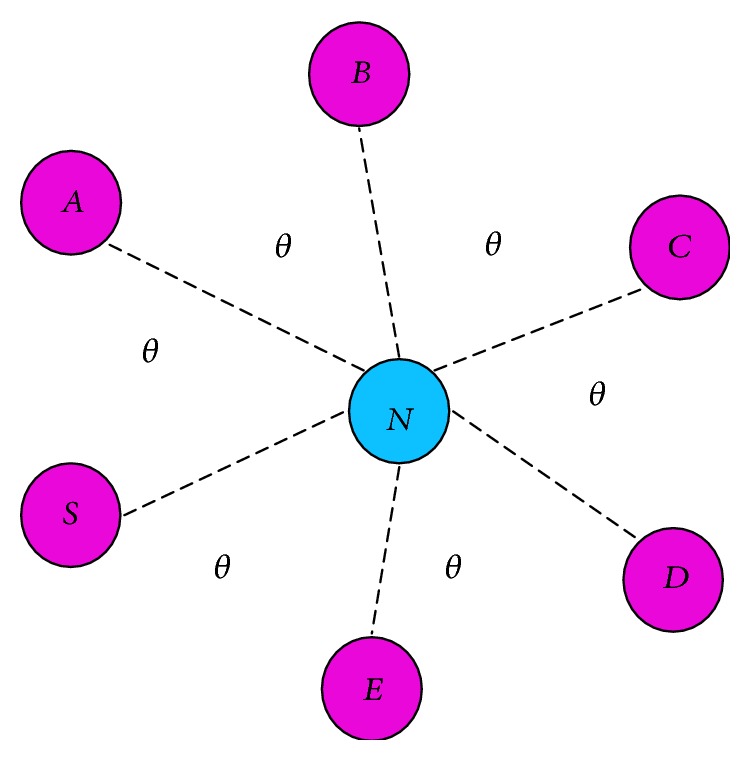
Next hop neighbor angle.

Figure 4 shows that the node *S* is a source, *N* is a next hop node, and *A*, *B*, *C*, *D*, and *E* are node *N*'s neighbors. Source collects the angles that node *N* makes with *N*'s neighbors (shown as *θ* in Figure 4). Note that *θ* is an arbitrary value and varies from one node to another unlike in Figure 4.

**Figure 5:**
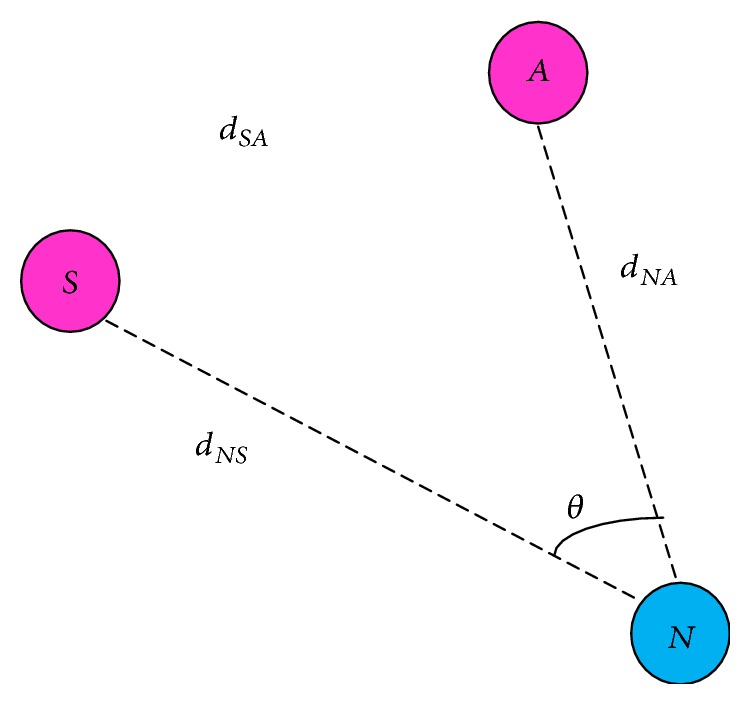
Individual neighbor angle.

Figure 5 shows the nodes *S* and *A* belonging to *N*'s neighbor list. In triangle, the distance of *d*_*NS*_, *d*_*SA*_, and *d*_*NA*_ is obtained from the RSS values of the acknowledgement received from node *S* and node *A*. The angle *∠SNA* is represented as *θ*. The value of *θ* is computationally obtained by the equation given below in (2).

The distance is measured by

(1) $$\begin{array}{c} d=\sqrt{{\left(x-x_{1}\right)}^{2}+{\left(y-y_{1}\right)}^{2}}, \end{array}$$

where *x*_1_ and *x*_2_ are the coordinates of the two nodes between distances. The angle *θ* is calculated based on

(2) $$\begin{array}{c} \theta =\arccos\left[\;\frac{b^{2}+c^{2}-a^{2}}{2bc}\;\right], \end{array}$$

where *a* → *d*_*SA*_, *b* → *d*_*NS*_, *c* → *d*_*NA*_.

**Figure 6:**
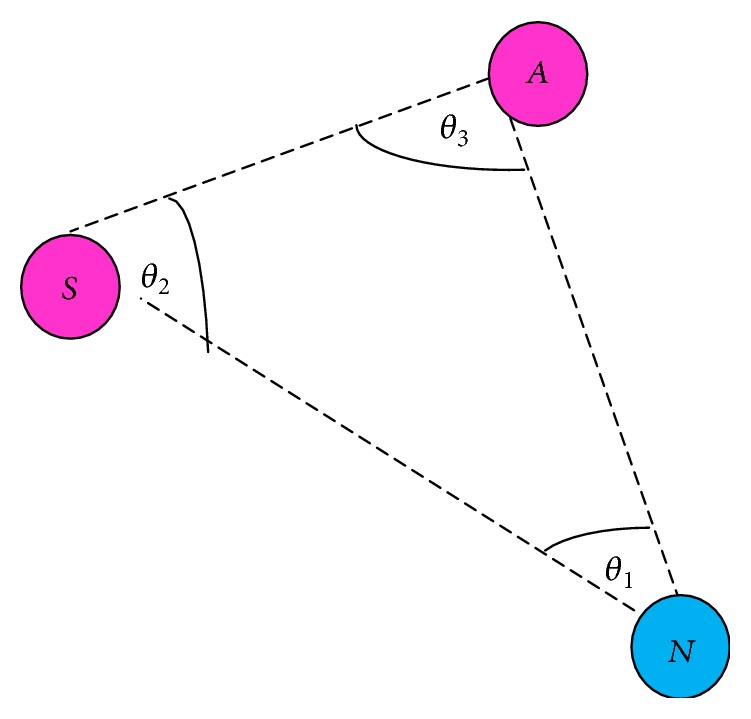
Interior angles.

Similarly, the algorithm computes every neighbor angle of the node *N*. The source stores every neighbor node angle in a table. Source *S*, next hop node *N*, common neighbor of *S* and *N* = node *A* form a triangle. The three interior angles are discovered and added up.  Figure 6 shows that the *θ*_1_, *θ*_2_, and *θ*_3_ are three interior angles. Mathematically, the addition of all three interior angles is 180°, where *∠SNA* = *θ*_1_, *∠ASN* = *θ*_2_, and *∠NAS* = *θ*_3_:

(3) ∠SNA+∠ASN+∠NAS=180°,θ1+θ2+θ3=180°

In Figure 5, the angle computed from the RSS for *∠SNA* = *θ* replaces the interior angle *∠SNA* = *θ*_1_. Now according to the property of a triangle,

(4) $$\begin{array}{c} \theta +\theta_{2}+\theta_{3}=180^{{}^{\circ}}. \end{array}$$

If the sum of the angles in the expression (4) adds up to make 180 degrees, then the nodes that make up the triangle are proved to be legitimate nodes. Hence, the source selects the next hop node only if the node is legitimate according to this method. Otherwise, one among the three nodes forming the triangle is said to be faulty and Level 1 test fails here. The source needs to identify which node is a precarious node and for that a novel Context Free Grammar verification method is proposed in the following section.

### 4.3. CFG Based Node Verification

The CFG based node verification (Level 2 test) is only performed when the angle based detection mechanism detects a faulty node. Generally, all nodes of the network have a mapping variable. The Leftmost and Rightmost derivations are obtained based on this mapping variable. The source *S* computes the leftmost derivation and the next node *N* computes the rightmost derivation. Finally, the source checks whether these derivations are equal or not. If it is equal, then node is legitimate; otherwise, the node is precarious.

#### 4.3.1. Leftmost and Rightmost Derivation

A leftmost derivation chooses the leftmost nonterminal to expand and the right most derivation chooses the rightmost nonterminals to expand. Every node is assigned a mapping function in the proposed system and the leftmost and rightmost derivations are obtained based on this mapping function.

**Figure 7:**
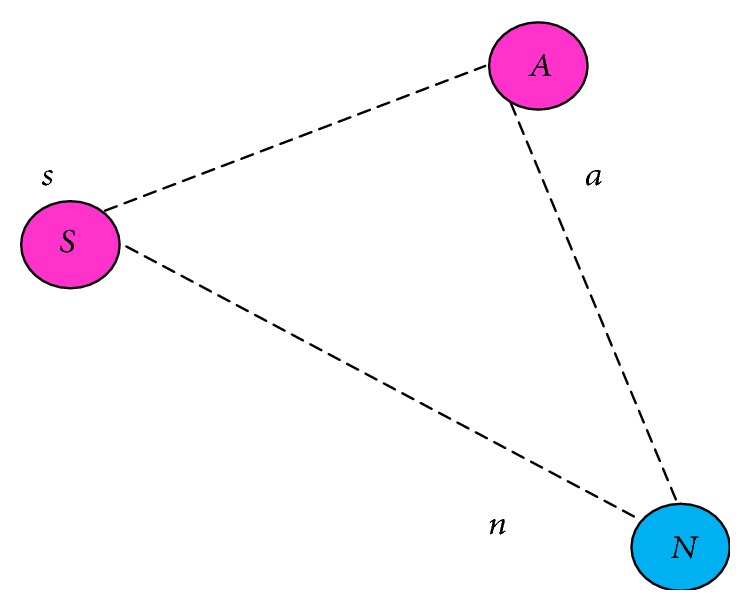
Mapping function.

In Figure 7, *S* is a source, *N* is a next hop, and *A* is a common node. Let node *a* be a mapping function of *A*, let *n* be a mapping function of *N*, and let *s* be a mapping function of *S*; these mapping functions will be applied to the CFG. The source checks nodes *A* and *N*, which node of left most and right most derivation is equal so that it can be selected as the next forwarding node. For example, the leftmost and rightmost derivation of nodes *S* and *N* is given below.

Consider Figure 7: the derivation is obtained among nodes *S*, *A*, and *N*. The mapping functions are *s*, *a*, and *n*. The leftmost and rightmost derivation between *S* and *N* is given below.

Given the grammar, *G* = ({*S*}, {*s*, *n*}*R*, *S*).

Consider the grammar *G* with production:

(5) $$\begin{array}{c} R=\left\{\;S⟶sSSn,S⟶a,S⟶n,S⟶s\;\right\}. \end{array}$$

The leftmost derivation of source and next hop is given below:

(6) $$\begin{array}{c} S\overset{LM}{⟹}sSSn\overset{LM}{⟹}ssSn\overset{LM}{⟹}ssnn. \end{array}$$

Rightmost derivation of source and next hop is the following:

(7) $$\begin{array}{c} S\overset{RM}{⟹}sSSn\overset{RM}{⟹}sSnn\overset{RM}{⟹}ssnn. \end{array}$$

**Figure 8:**
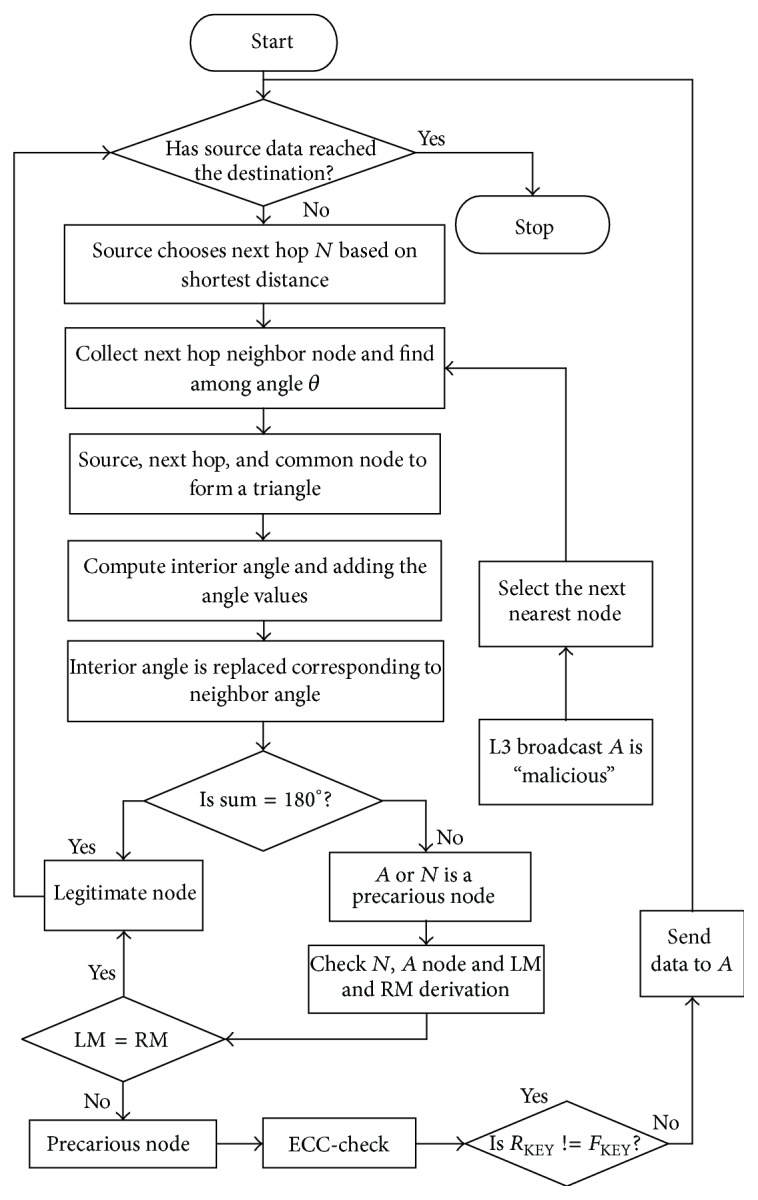
Flowchart of ACFG scheme.

The leftmost derivation is obtained based on the mapping function and stores the string value in a table. Next node/common node is derived by the rightmost derivation. If the source checks leftmost and right most derivations are equals, that node is legitimate; otherwise it is a precarious node. The algorithm used to design the ACFG mechanism is given in Algorithm 1 that corresponds to the various steps illustrated in the flowchart of Figure 8.

### 4.4. Secure Data Transmission Using Reinforcement

**Figure 9:**
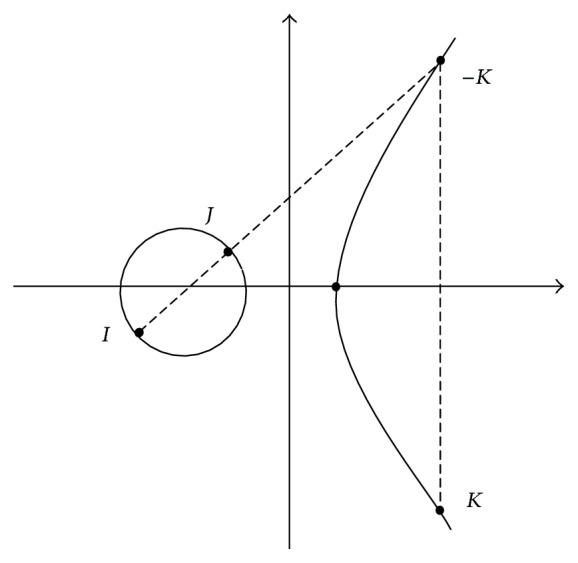
Elliptic Curve representing source and next node coordinates *I* and *J*.

The reinforcement action of this scheme is to publish that a node is malicious to all other nodes so that the node can be excluded from the communication process. This comes under Level 3 (L-3) action of the proposed mechanism. To incorporate this, there is need to confirm that a node is totally illegitimate before broadcasting its label “malicious” to all nodes. Therefore, the node that fails the CFG test is further examined using the elliptical curve cryptography technique using the Weierstrass elliptic function. Consider the coordinate points of the source and the next node *N* to be *I* and *J*, respectively; there is another point *K* which forms a straight line as illustrated in Figure 9.

The Weierstrass elliptic function is defined

(8) y2=x3+px+qI+J=Kwhere  I≠J,  ∀I,J∈E,

where (*x*_*I*_, *y*_*I*_) and (*x*_*J*_, *y*_*J*_) and (*x*_*K*_, *y*_*K*_) are the coordinates of the *I*, *J*, and *K* points forming an elliptic curve. Therefore, the coordinates (*x*_*K*_, *y*_*K*_) can be obtained from the following expressions:

(9) xK=β2−xI−xJ,yK=βxI−xK−yI,

where *β* = (*y*_*J*_ − *y*_*I*_)/(*x*_*J*_ − *x*_*I*_).

The commutative property of this function states that

(10) $$\begin{array}{c} I+J=J+K. \end{array}$$

The working of this Level 3 test for publishing the node as malicious is given by Algorithm 2. The algorithm shows that the node estimates and sends L3_REQ_ to the next node *N*. Meanwhile the *F*_KEY_ is estimated using (11) at the source end. The next node replies to the L3_REQ_ with *R*_KEY_ using (12):

(11) FKEY=I+J,

(12) RKEY=J+K.

The source node *S* compares its *F*_KEY_ with the *R*_KEY_ to conclude that the next node *N* is a malicious node. When *F*_KEY_ is not same as *R*_KEY_ then the node is published as malicious to all other nodes and the next nearest node is considered for communication. Algorithm 2 is a part of the main Algorithm 1.

## 5. Performance Metrics

Five metrics are assessed in the simulation analysis of the network. They are Packet Delivery Ratio, Packet Loss Ratio, Throughput, Delay, and Detection Rate.

### 5.1. Packet Delivery Rate

Packet Delivery Rate (PDR) is the ratio of the total number of packets successfully delivered to the total packets sent. It is obtained from (13) below, where *n* represents the total number of nodes in the networks:

(13) $$\begin{array}{c} PDR=\frac{\sum_{0}^{n}PktsDelivered}{Time}. \end{array}$$

Here *PktsDelivered* is the number of packets received by the destination and *PktsSent* is the number of packets sent by the source.

### 5.2. Packet Loss Rate

Packet Loss Rate (PLR) is the ratio of the packets lost to the total packets sent, estimated by

(14) $$\begin{array}{c} PLR=\frac{\sum_{0}^{n}PktsLost}{Time}\;\left\{0\leq PLR\leq \infty \right\}. \end{array}$$

### 5.3. Throughput

Throughput is defined as the rate at data is successfully transmitted for every packet sent, evaluated by

(15) Throughput=∑0nPacketsReceived∗8Delay  in  mskbps0≤Throughput≤∞.

### 5.4. Delay

Delay is defined as the time difference between the current packets received and the previous packet received, evaluate by (16) below, where *n* is the number of nodes:

(16) Delay=∑0nPktRecvd  Time−PktSend  Timen0≤PDR≤∞.

### 5.5. Detection Ratio

In this paper, we observe the detection ratio and false detection ratio of AODV and ACFG routing protocols. The detection ratio and false detection ratio are defined as (17) follows:

(17) DR=DpnTpn0≤DR≤1,FDR=MpnTnn0≤FDR≤1,

where *D*_pn_ is the number of precarious node detected by one or more normal nodes, *T*_pn_ is the total number of precarious nodes, *M*_pn_ is the number of normal nodes misidentified as the precarious node by one or more normal nodes, and *T*_nn_ is the total number of normal node.

## 6. Experimental Results and Discussion

The Network Simulator-2 is used to study the performance of our precarious node detection and secure data transmission in MANETs. We apply the IEEE 802.11. MAC with channel data rate 10 Mbps. Remaining parameters are available in Table 1.

### 6.1. Mobility Analysis

**Figure 10:**
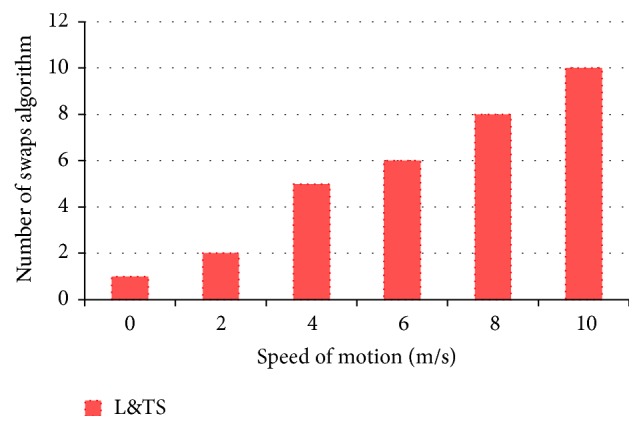
Effect of mobility in L&TS scheme.

Mobility in MANETs is a hindrance for the implementation of many security schemes since it affects the QoS of the system. The security of L&TS scheme increases as the mobility increases.  Figure 10 indicates mobility versus the number of swaps between the algorithms used. Greater the number of swaps, greater the security of the system. There is a tradeoff between QoS and security in this scheme.

### 6.2. Comparison of TMUR, L&TS, and ACFG with CBR Traffic Models

In order to validate the efficiency of the ACFG, we compare it with TMUR and L&TS protocol. The performance and metrics described in Section 5 are used here.

**Figure 11:**
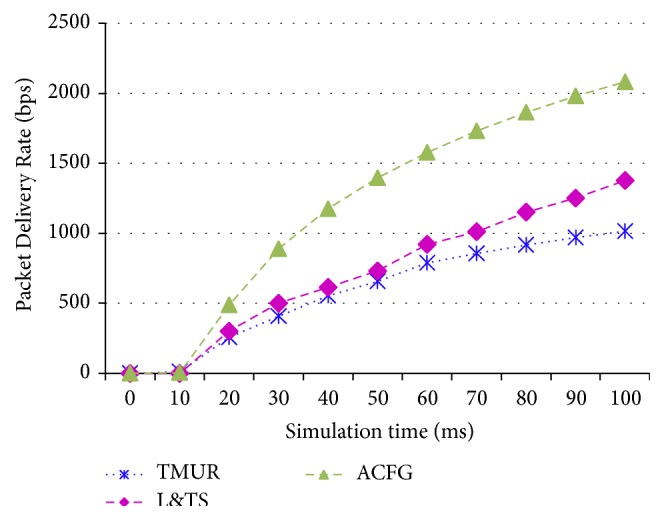
Packet Delivery Rate of TMUR, L&TS, and ACFG.

**Figure 12:**
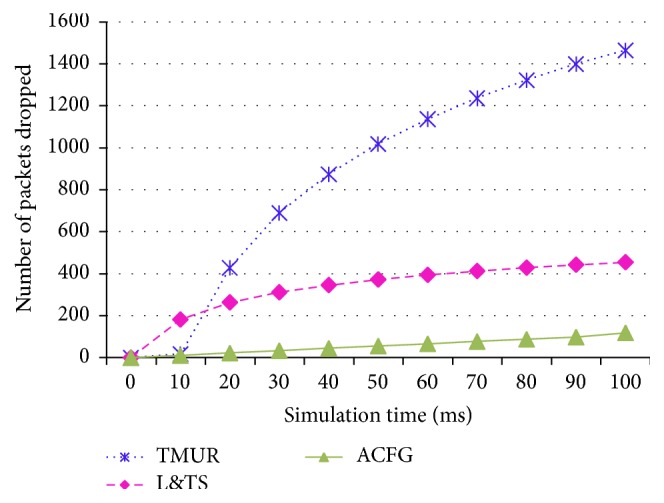
Packet Loss Rate of TMUR, L&TS, and ACFG.

Figures 11 and 12 show the Packet Delivery Rate and Packet Loss Rate of the TMUR, L&TS, and ACFG mechanisms, respectively. These two metrics are proportional to each other and indicate the successful communication among the mobile nodes in any MANET. Therefore, it can be observed from the graphs that L&TS performs better than TMUR and ACFG performs better than both the mechanisms due to secure communication and the three-level checks performed over the nodes in the MANET.

**Figure 13:**
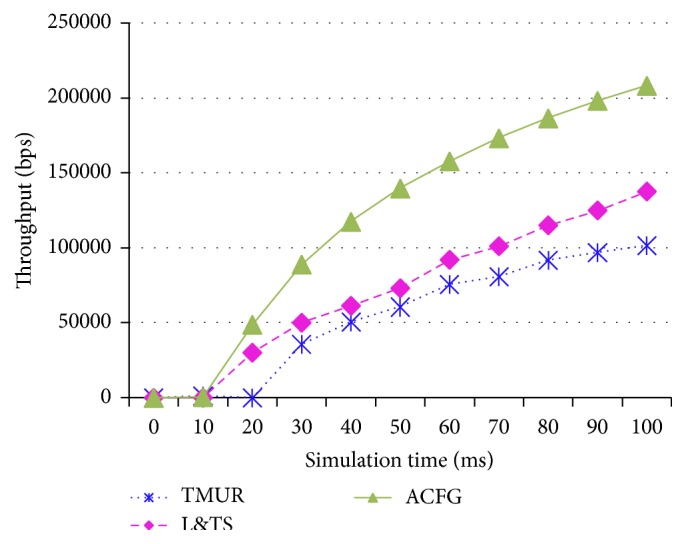
Throughput of TMUR, L&TS, and ACFG.

**Figure 14:**
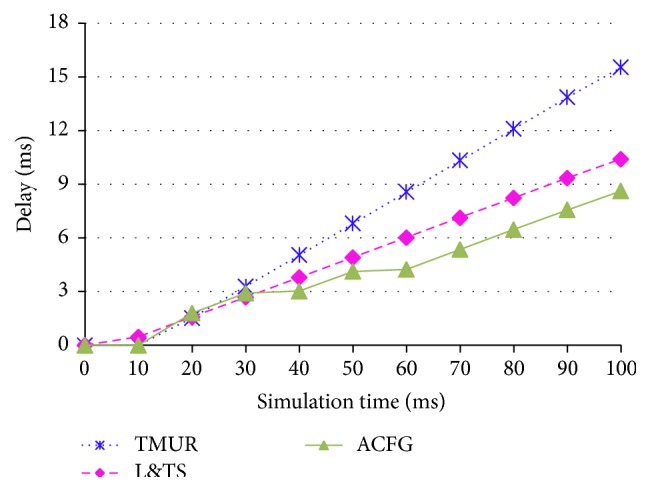
Delay of TMUR, L&TS, and ACFG.

Figure 13 shows the throughput obtained for the of TMUR, L&TS, and ACFG mechanisms. It can be observed that the maximum throughput obtained for ACFG is greater than the L&TS mechanism, which is in turn greater than the TMUR. Similarly, the delay observed in a MANET operated using the three protocols is also plotted in Figure 14. The ACFG mechanism shows the least delay compared to the L&TS that is also lower than the TMUR protocol. The malicious nodes present in the network obstruct the communication in the network and therefore the ACFG mechanism both avoids and detects the best performance among the three protocols.

**Figure 15:**
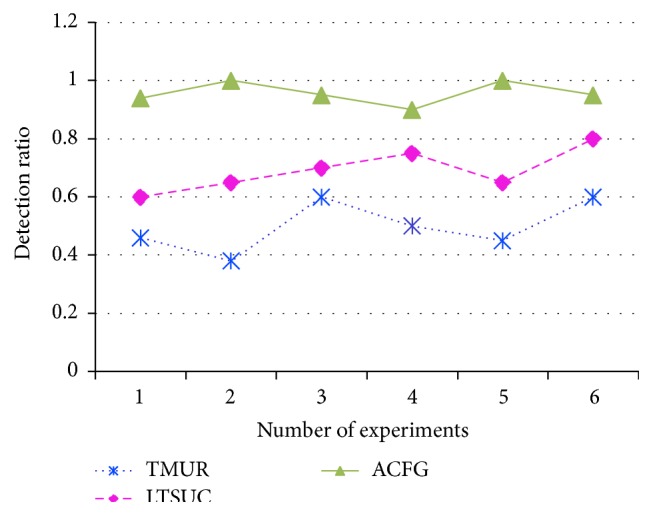
Detection ratio of TMUR, L&TS, and ACFG.

**Figure 16:**
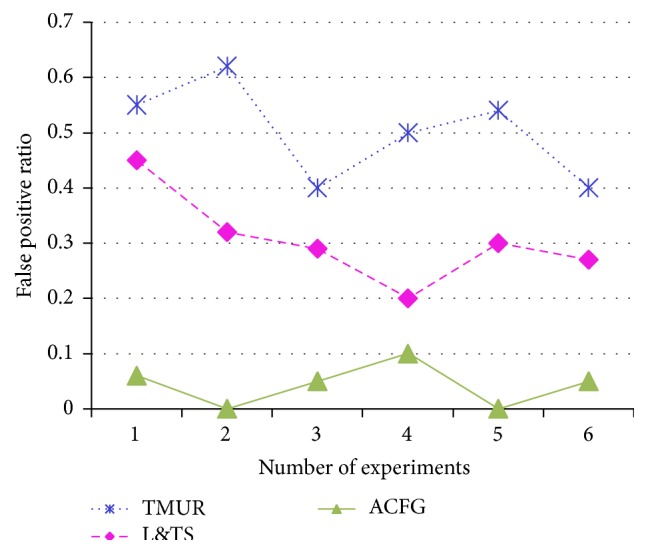
False positive ratio of TMUR, L&TS, and ACFG.

The detection ratio of TMUR, L&TS, and ACFG mechanisms is plotted in Figure 15. After modeling a 20% of the nodes as attack nodes, the three methods are tested whether they are able to efficiently identify and detect the malicious nodes. It can be observed from the figure that the detection rate of ACFG is greater than both L&TS and ACFG mechanisms. Also the false positive ratio is plotted for the three comparing mechanisms in Figure 16 to observe that the ACFG mechanism has the lowest false positive ratio.

### 6.3. Comparison of Throughput in CBR and TCP Models

The operation of the ACFG mechanism using both Constant Bit Rate (CBR) and Variable Bit Rate (VBR) traffic models are validated. UDP does not contain acknowledge packet (ACK) that permits the nonstop packet stream, as opposed to using TCP that acknowledges a set of packets calculated by using the TCP window size and Round Trip Time (RTT).

**Figure 17:**
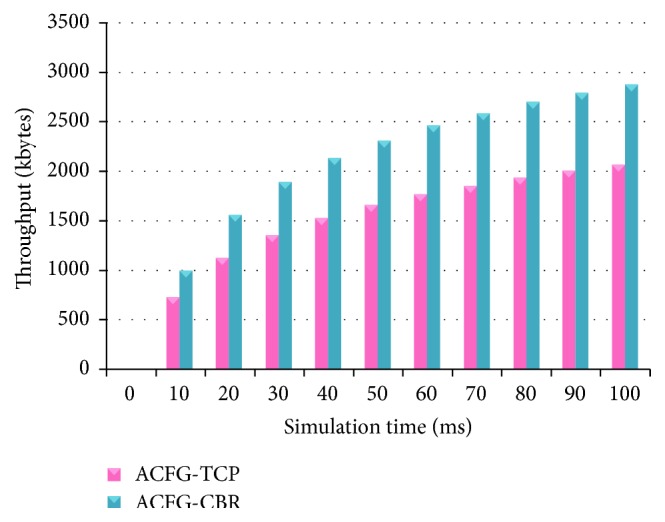
Throughput of ACFG with CBR and TCP models.

**Figure 18:**
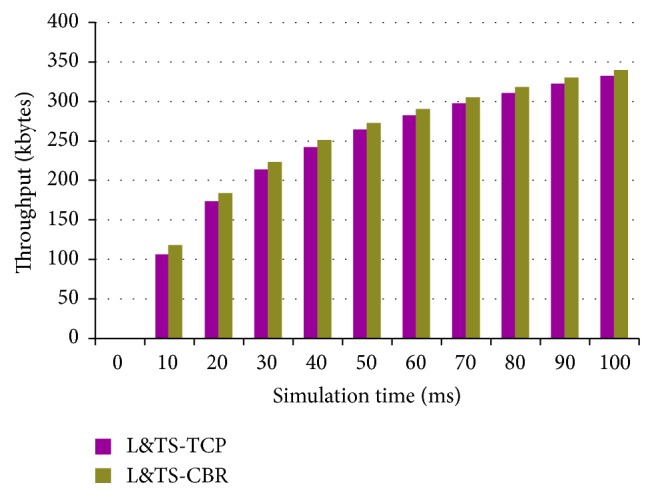
Throughput of L&TS with CBR and TCP models.

Figure 17 shows the throughput of ACFG with both CBR and TCP models. ACFG works better with CBR traffic model when compared with the TCP models.  Figure 18 shows the throughput of the L&TS for CBR and TCP traffic models. The CBR generates slightly longer throughput than TCP. The CBR traffic model is better when compared to the TCP.

### 6.4. Comparison of Throughput against Node Mobility

**Figure 19:**
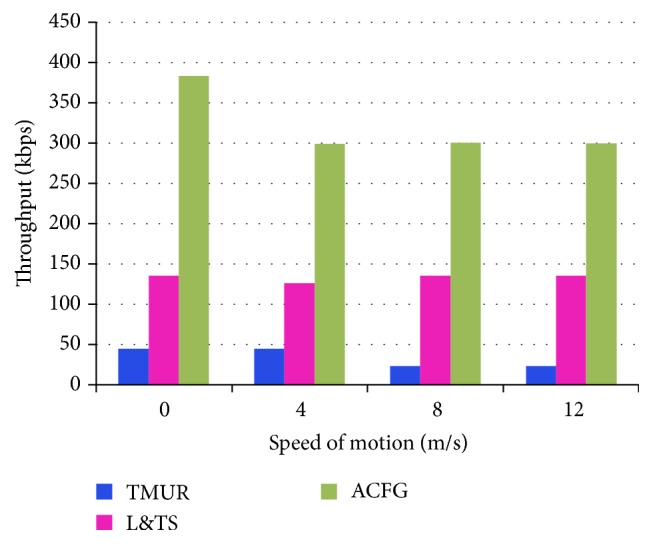
Throughput against node mobility.

In Figure 19, it can be observed that the TMUR suffers more from the speed of motion compared with the ACFG and L&TS. The security in TMUR does not vary under mobility because the mobility is increased when security algorithm is increased. Hence, the throughput does not change based on the mobility. However, ACFG obtains better throughput rate, compared to TMUR.

### 6.5. Comparison of Throughput against Number of Nodes

**Figure 20:**
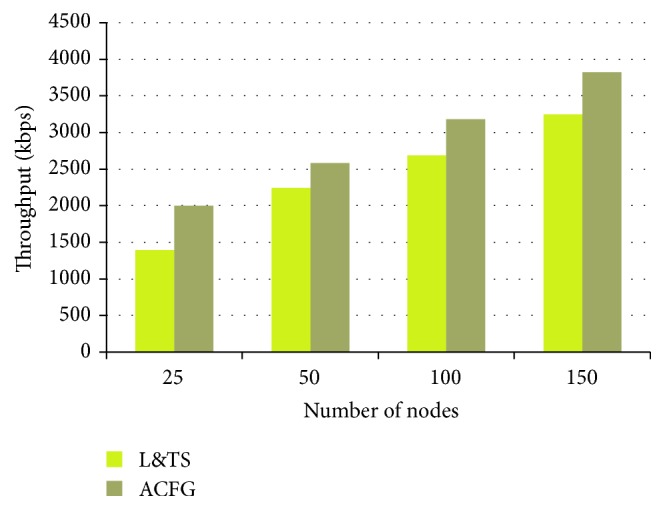
Throughput against number of nodes.

According to analysis performed, the scalability of the proposed mechanisms is achieved by obtaining the throughput varying the number of nodes from 25 to 150 within an area of 1000 × 1000 m simulation field. From Figure 20, it has been observed that ACFG performs better than L&TS with average throughput crossing 3000 kbps.

A summary of the results shown in Figures 10–20 is tabulated in Table 2. The various parameters measured for TMUR, L&TS, and ACFG are tabulated to analyze the overall improvements.

The simulation results show that the dynamic estimation of the metrics improves throughput by 26% in L&TS when compared to the TMUR. ACFG achieves 33% and 51% throughput increase when compared to L&TS and TMUR mechanisms, respectively.

## 7. Conclusions

In this paper, we have proposed two new mechanisms for incorporating secure communication in MANETs. This paper contains two strategies: Location and Trust-based secure routing (L&TS) as well as Angle and CFG based precarious node detection (ACFG) with secure data transmission in MANETs. L&TS method uses various cryptography algorithms based on distance and includes trust based routing. ACFG method isolates the precarious node based on the Angle and Context Free Grammar and secures data transmission using the SHA-1 algorithm. The simulation results evaluate that both the ACFG and L&TS mechanisms offer improved throughput and reduced delay, more so the ACFG. In future works, we intend to investigate the precarious node detection in Cognitive Networks.

## Figures and Tables

**Algorithm 1:**
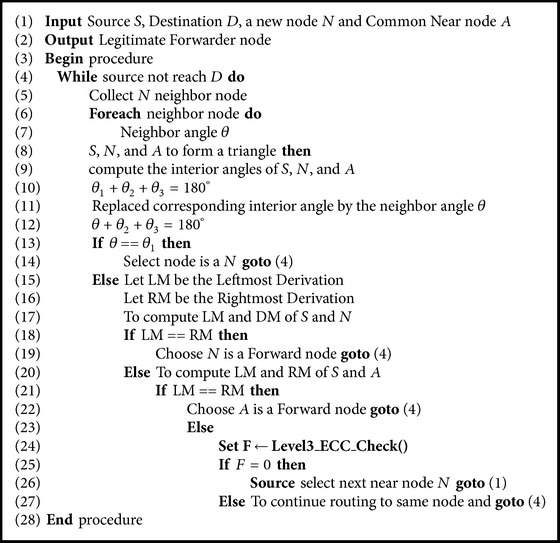


**Algorithm 2:**
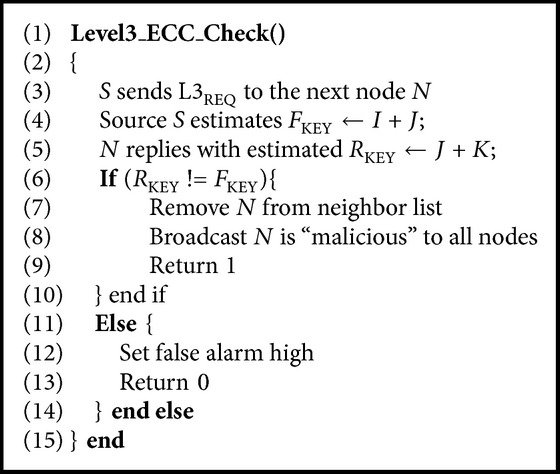


**Table 1:** Simulation parameters.

Parameter	Value
Simulation area	1000 × 1000 m
Number of nodes	30
Channel	WirelessPhy
Channel data rate	10 Mbps
Radio propagation model	TwoRayGround
Antenna type	Omni Antenna
Traffic models	CBR/TCP
CBR interval	1.0 ms
Communication model	UDP
Mobility model	Random way point
Simulation time	100

**Table 2:** Comparison of results.

Parameters	TMUR	L&TS	ACFG
Packet Delivery Rate (bps)	1015	1376	2083
Packet Loss Rate (packets)	1465	454	118
Throughput (bps)	101599	137604	208319
Delay (ms)	15.5	10.4	8.6
Average detection ratio	0.49	0.69	0.95
Average false positive ratio	0.50	0.30	0.04

## References

[1] Dahill B., Levine B., Royer E., Shields C. (2000). A secure routing protocol for ad hoc networks.

[2] Papadimitratos P., Haas Z. J. Secure routing for mobile ad hoc networks.

[3] Zheng Y., Peng Z., Teemupekka V. (2006). *Trust Evaluation Based Security Solution in Ad Hoc Networks*.

[4] Joshi K. C., Pant D. Security of mobile Ad-hoc networks with five layer security architecture.

[5] Saleh M., Dong L. (2013). Real-time scheduling with security enhancement for packet switched networks. *IEEE Transactions on Network and Service Management*.

[6] Liu W., Yu M. (2014). AASR: authenticated anonymous secure routing for MANETs in adversarial environments. *IEEE Transactions on Vehicular Technology*.

[7] Abdelhakim M., Lightfoot L. E., Ren J., Li T. (2014). Distributed detection in mobile access wireless sensor networks under byzantine attacks. *IEEE Transactions on Parallel and Distributed Systems*.

[8] Bouassida M. S., Guette G., Shawky M., Ducourthial B. (2009). Sybil nodes detection based on received signal strength variations within VANET. *International Journal of Network Security*.

[9] Bhuvaneswari P. T. V., Karthikeyan S., Jeeva B., Prasath M. A. An efficient mobility based localization in underwater sensor networks.

[10] Heiko W., Schiller J. Distance-based distributed multihop localization in mobile wireless sensor networks. http://page.mi.fu-berlin.de/eke/will09FGSN.pdf.

[11] Karma A., Choudhary J. (2014). MPLI: a novel modified parametric location identification for AODV in MANET. *International Journal of Computer Applications*.

[12] Zhu Z., Guan W., Liu L., Li S., Kong S., Yan Y. A multi-hop localization algorithm in underwater wireless sensor networks.

[13] Li J., Halder B., Stoica P., Viberg M. (1995). Computationally efficient angle estimation for signals with known waveforms. *IEEE Transactions on Signal Processing*.

[14] Venkata C., Singhal M. Angular routing protocol for mobile ad-hoc networks.

[15] Boukerche A., Oliveira H. A. B. F., Nakamura E. F., Loureiro A. A. F. (2007). Localization systems for wireless sensor networks. *IEEE Wireless Communications*.

[16] Shih T.-F., Yen H.-C. (2008). Location-aware routing protocol with dynamic adaptation of request zone for mobile ad hoc networks. *Wireless Networks*.

[17] Suri P. K., Soni M. K., Tomar P. (2011). Framework for location based power aware routing in MANET. *IJCSI International Journal of Computer Science Issues*.

[18] Cheng L., Wu C., Zhang Y., Wu H., Li M., Maple C. (2012). A survey of localization in wireless sensor network. *International Journal of Distributed Sensor Networks*.

[19] Chamundeeswari R. M., Sumathi P. (2014). Efficient detection of intrusion using inner and outer boundary models with transductive learning concept in mobile Adhoc network. *International Journal of Scientific & Engineering Research*.

[20] Singh A., Dagon D., Dos Santos A. L. M. (2004). Authentication protocols making use of context free grammar: guessing strings.

[21] Geyik S. C., Szymanski B. K. Event recognition in sensor networks by means of grammatical inference.

[22] Jadhav S., Kulkarni U. L. (2014). Natural language database interface with probabilistic context free grammar. *IJRIT: International Journal of Research in Information Technology*.

